# Syncope in Patients with Cardiac Pacemakers

**DOI:** 10.21470/1678-9741-2020-0076

**Published:** 2021

**Authors:** Eduardo Arrais Rocha, Gisele Schineider Cunha, Aline Bezerra Tavares, Antônio Brazil Viana Júnior, Ana Rosa Pinto Quidute, Francisca Tatiana Moreira Pereira, Marcelo de Paula Martins Monteiro, Maria Eduarda Quidute Arrais Rocha, Camila Rabelo Ferreira Gomes, Carlos Roberto Martins Rodrigues Sobrinho

**Affiliations:** 1 Department of Clinical Medicine, Federal University of Ceará, Fortaleza, Ceará, Brazil.; 2 Department of Physiology and Pharmacology, Federal University of Ceará, Fortaleza, Ceará, Brazil.; 3 University of Fortaleza, Fortaleza, Ceará, Brazil.

**Keywords:** Syncope, Pacemaker, Artificial, Cardiac Conduction System Disease, Surveys and Questionnaires, Attention

## Abstract

**Introduction:**

It is challenging to diagnose syncope in patients with pacemakers. Because these patients have increased morbidity and mortality risks, they require immediate attention to determine the causes in order to provide appropriate treatment. This study aimed to investigate the causes and predictive factors of syncope as well as the methods used to diagnose syncope in cardiac pacemaker patients.

**Methods:**

Patients with pacemakers implanted owing to sinus node disease or atrioventricular block were evaluated with standardized questionnaires, endocavitary electrograms, and other tests based on the suspected causes of syncope. Mann-Whitney U tests were used to analyze continuous variables and Chi-squared or Fisher’s exact tests were used for categorical variables. Logistic regression was used for multivariate analyses. Statistical significance was *P*<0.05.

**Results:**

The study included 95 patients with pacemakers: 47 experienced syncope in the last 12 months and 48 did not. Of the 100 documented episodes of syncope, 48.9% were vasovagal syncopes, 17% had cardiac-related causes, 10.6% had unknown causes, and 8.5% had pacemaker failure. The multivariate analysis showed that a New York Heart Association (NYHA) Functional Class II was a significant factor for developing syncope (*P*<0.01).

**Conclusion:**

While the most common type of syncope in pacemaker patients was neurally mediated, it is important to perform detailed evaluations in this population as the causes of syncope can be life-threatening. The best diagnostic methods were stored electrogram analysis and the tilt table test. NYHA Functional Class II patients were found to have a higher risk for syncope.

**Table:** 

Abbreviations, acronyms & symbols	
**AF**	**= Atrial fibrillation**
**CI**	**= Confidence interval**
**ECG**	**= Electrocardiograms**
**EGM**	**= Electrogram**
**IES**	**= Invasive electrophysiological study**
**LVDD**	**= Left ventricular diastolic diameter**
**LVEF**	**= Left ventricular ejection fraction**
**NYHA**	**= New York Heart Association**
**OH**	**= Orthostatic hypotension**
**OR**	**= Odds ratio**
**PM**	**= Pacemaker**
**SVT**	**= Sustained ventricular tachycardia**
**VF**	**= Ventricular fibrillation**

## INTRODUCTION

Syncope is a transient, self-limited loss of consciousness due to cerebral hypoperfusion characterized by a short, sudden loss of postural tone with no permanent neurological damage^[1,2]^. It is difficult to diagnose the causes of syncope, especially in pacemaker (PM) patients. These patients require immediate attention to determine the causes and to provide appropriate treatment because they have higher risks of morbidity and mortality, and they often present with several associated comorbidities^[3,4]^. The causes can be complex and may have a host of potential diagnoses. Approximately 30% of syncopal episodes remain unexplained even after an extensive investigation^[5]^. Syncope can be caused by PM or electrode malfunction as well as other causes, including ventricular tachycardia, in heart disease patients^[3,5]^.

Autonomic dysfunction can occur in PM patients because the device may interfere only with the cardioinhibitory component of neurally-mediated syncope by preventing bradycardia; however, vasodilation, which is the main symptom in most patients with vasovagal syncope, is still present^[6,7]^. Many elderly patients with associated neurological pathologies have a PM and present with dysautonomia involving varying degrees of orthostatic intolerance and syncope. These patients are often on multiple drugs, and polypharmacy may also contribute to syncope.

Several complementary methods are used to investigate the causes of syncope, including invasive methods^[8]^, such as electrophysiological (EEF) study, or noninvasive methods, such as the electrocardiogram, Holter monitor, tilt table test, echocardiogram, and exercise stress test. However, these methods usually have a low sensitivity for a proper diagnosis. The misuse of these methods may also increase costs, prolong investigation time, and lead to further confusion about the diagnosis. Several long-term cardiac monitoring methods, such as external or implantable loop recorders, have revolutionized diagnosing the causes of syncope. Modern cardiac PMs have sophisticated diagnostic functions, including the endocavitary electrogram (EGM), which records both symptomatic and asymptomatic arrhythmic events. Precise analysis of these records can either diagnose or eliminate several causes of syncope.

Because of the diagnostic complexity and lack of existing studies on syncope in cardiac PM patients, our study aimed to investigate the main causes and predictors of syncope as well as complementary methods used to diagnose the causes of syncope in these patients.

## METHODS

### Population and Study Design

This is a prospective cohort study with participants recruited from the outpatient clinic for arrhythmias and PM at the Clinical Hospital of the Federal University of Ceará, Brazil, between May 2015 and January 2018. Patients with uni- or bicameral PMs with a capacity for EGM implanted in the last 10 years for sinus node dysfunction or atrioventricular block were included. Patients referred to the arrhythmia clinic for syncope or attending a routine follow-up appointment were recruited for the study.

Patients with an implantable cardioverter defibrillator and multisite or biventricular PM were excluded. Those who had at least one episode of syncope in the last 12 months were assigned to the case group, and those with no history of syncope were assigned to the control group. Quarterly follow-ups were performed in the first year or, in cases of recurrent syncope, were performed earlier. Members of the control group were matched with members of the case group weekly.

Patients with structural heart disease included those with heart failure (as well as those with preserved ejection fraction or left ventricular dysfunction), moderate-to-severe left ventricular hypertrophy, known coronary artery disease, previous myocardial revascularization, previous stenting, or moderate-to-severe valvulopathy.

Investigative protocols included obtaining a detailed history of the episodes of syncope with standardized questionnaires performed by cardiologists specializing in arrhythmias along with a physical examination including evaluating for orthostatic hypotension (OH), PM testing, EGM analysis with myopotential inhibition tests, standard ECG, and echocardiograms. After clinical evaluation, additional tests such as 24-hour Holter monitoring, seven-day Holter monitoring, tilt table testing, carotid sinus hypersensitivity testing, electrophysiological studies, or neurological examinations were requested if symptoms related to the tests were clinically suspected. OH was included in the dysautonomia/vasovagal group. The authors adhered to the European Society of Cardiology Guidelines for the Diagnosis and Management of Syncope^[1]^.

This study was approved by the ethics committee of the hospital where it was conducted. All participants or their legal representatives signed the terms of free and informed consent.

### Statistical Analysis

Data was verified by the Shapiro-Wilk test. Continuous variables were analyzed using Mann-Whitney U tests, and categorical variables were analyzed using Chi-squared tests or Fisher’s exact tests when appropriate. Multivariate logistic regression analysis was performed, including variables with *P*<0.10 in a univariate analysis. Statistical significance was set at *P*<0.05. The software used for analysis was R Open 3.5.1 (Microsoft Corp, Redmond, Washington, United States of America).

## RESULTS

The study included 95 PM patients: 47 had at least one episode of syncope in the last 12 months, and 48 had no episodes (Table 1). The mean follow-up period was 18.5 months. There were three deaths in the group with syncope and none in the group without it. We recorded 100 episodes of syncope in 47 individuals. Two events were recorded in 14 patients, and 11 patients experienced more than three events.

**Table 1:** Characteristics of study patients (n=95).

Variables		Syncope	Without syncope	*P*-value
Gender	Male	23 (48.9%)	26 (54.2%)	0.610^c^
	Female	24 (51.1%)	22 (45.8%)	
Age		82 (72 - 87)	78.5 (71.5 - 85)	0.406^b^
> 3 comorbidities		28 (59.6%)	17 (35.4%)	0.018^c^
NYHA class^a^	I	18 (38.3%)	43 (89.6%)	0.001^d^
	II	23 (48.9%)	4 (8.3%)	
	III	6 (12.8%)	1 (2.1%)	
Pacemaker dependence		16 (34.0%)	4 (8.3%)	0.002^c^
Structural cardiopathy		28 (59.6%)	20 (41.7%)	0.081^c^
Postural hypotension		12 (25.5%)	1 (2.1%)	0.001^c^
LVEF		59 (51 - 63)	59 (56 - 62)	0.774^b^
LVDD (mm)		51 (47 - 55)	48.5 (47 - 54.5)	0.266^b^

CI=confidence interval; NYHA=New York Heart Association; OR=odds ratio

a There were no NYHA class IV patients

b Mann-Whitney U test

c Pearson chi-square test

d Fisher's exact test

Comorbidities - presence of three or more of the following: fragility syndrome, previous stroke, diabetes, heart failure, chronic obstructive pulmonary disease, and vasculopathy; structural cardiopathy - left ventricular dysfunction, moderate to severe left ventricular hypertrophy, moderate to severe coronary artery disease, or valvopathy. Significance was set as *P*<0.05. Data shown in n (%) and median (25^th^ percentile - 75^th^ percentile)

The causes of syncope in PM patients were: vasovagal/dysautonomia (48.9%), cardiac-related (17%), unknown (10.6%), PM failure (8.5%), neurologic (8.5%), and other reasons (6.3%, *e.g*., two for hypoglycemia and one for psychogenic cause). In the group with syncope, 29.7% required hospitalization, 10.6% had physical trauma due to syncope, 68% had prodromes, 86.3% reported syncope prior to PM implantation, 13.7% had postural hypotension, and 18.9% had recurrent episodes. In patients with syncope, 95% had previously presented with episodes of pre-syncope.

Causal factors were identified in 40.4% of patients with syncope as follows: infection, gastroenteritis, postoperative state, rapid change of position, gastric bleeding, wrong insulin dose, and constipation with an increased effort to evacuate. These situations were found while reviewing clinical histories and considered plausible explanations for the onset of neurally-mediated syncope in PM patients.

In patients with cardiac-related causes of syncope (17%), three were related to high rate atrial fibrillation (AF), four related to sustained ventricular tachycardia (SVT), and one related to ventricular fibrillation (VF), which occurred during an acute myocardial infarction. The neurologic causes (8.5%) included three patients with seizures and one related to a transient ischemic attack.

In the group with differing causes, two experienced loss of consciousness related to hypoglycemic episodes and one for psychogenic cause (Table 2). The results of univariate and multivariate analyses related to predictive factors for syncope in PM patients are presented in Tables 3 and 4.

**Table 2:** Causes of syncope in pacemaker patients.

Causes	Incidence, n (%)
Vasovagal/dysautonomia	23 (48,9)
Cardiac	8 (17)
Unknown	5 (10,6)
Pacemaker failure	4 (8,5)
Neurologic*	4 (8,5)
Other reasons**	3 (6,3)
Total patients	47 (100)

In total, 100 episodes of syncope were recorded in 47 individuals. Fourteen patients had two events, and 11 experienced more than three. There were no different causes of syncope in the same patient. Patients with neurologic and other reasons did indeed not experience syncope, but rather transient loss of consciousness based on the Guidelines of Syncope^[1,2]^

* Three patients with epileptic seizures and one with transient ischemic attack

** Two patients had loss of consciousness because of hypoglycemia and one for psychogenic cause

**Table 3:** Significance of the variables associated with the occurrence of syncope in patients with pacemaker (n=95).

Variable	Univariate analysis
Postural hypotension	*P*=0.001
Pacemaker dependence	*P*=0.02
NYHA class II	*P*<0.01
NYHA class III	*P*<0.01
Structural cardiopathy	*P*=0.03
≥ 3 comorbidities	*P*=0.01

NYHA=New York Heart Association Comorbidities - presence of three or more of the following: fragility syndrome, previous stroke, diabetes, heart failure, chronic obstructive pulmonary disease, and vasculopathy; structural cardiopathy - left ventricular dysfunction, moderate to severe left ventricular hypertrophy, moderate to severe coronary artery disease, or valvopathy. Significance was set as *P*<0.05.

**Table 4:** Multivariate logistic regression analysis of variables of syncope occurrence in patients with pacemaker.

Variables		OR	95% CI OR	*P*-value
≥ 3 comorbidities		0.994	(0.320 - 3.08)	0.992
Structural cardiopathy		1.149	(0.376 - 3.51)	0.808
NYHA Functional Class	I	Reference		
	II	9.035	(2.319- 35.19)	0.002
	III	7.286	(0.639- 83.13)	0.110
Pacemaker dependence		3.565	(0.841- 15.11)	0.084
Postural hypotension		9.527	(0.992- 91.51)	0.051

NYHA=New York Heart Association; OR=odds ratio Comorbidities - presence of three or more of the following: fragility syndrome, previous stroke, diabetes, heart failure, chronic obstructive pulmonary disease, and vasculopathy; pacemaker dependence - pacemaker patients without rhythm after programming the lower frequency of 30 beats during 5 seconds; structural cardiopathy - left ventricular dysfunction, moderate to severe left ventricular hypertrophy, moderate to severe coronary artery disease, or valvopathy. Significance was set as *P*<0.05. Hosmer-Lemeshow test *P*=0.568/Nagelkerke R^2^ = 0.435

**Fig. 1:**
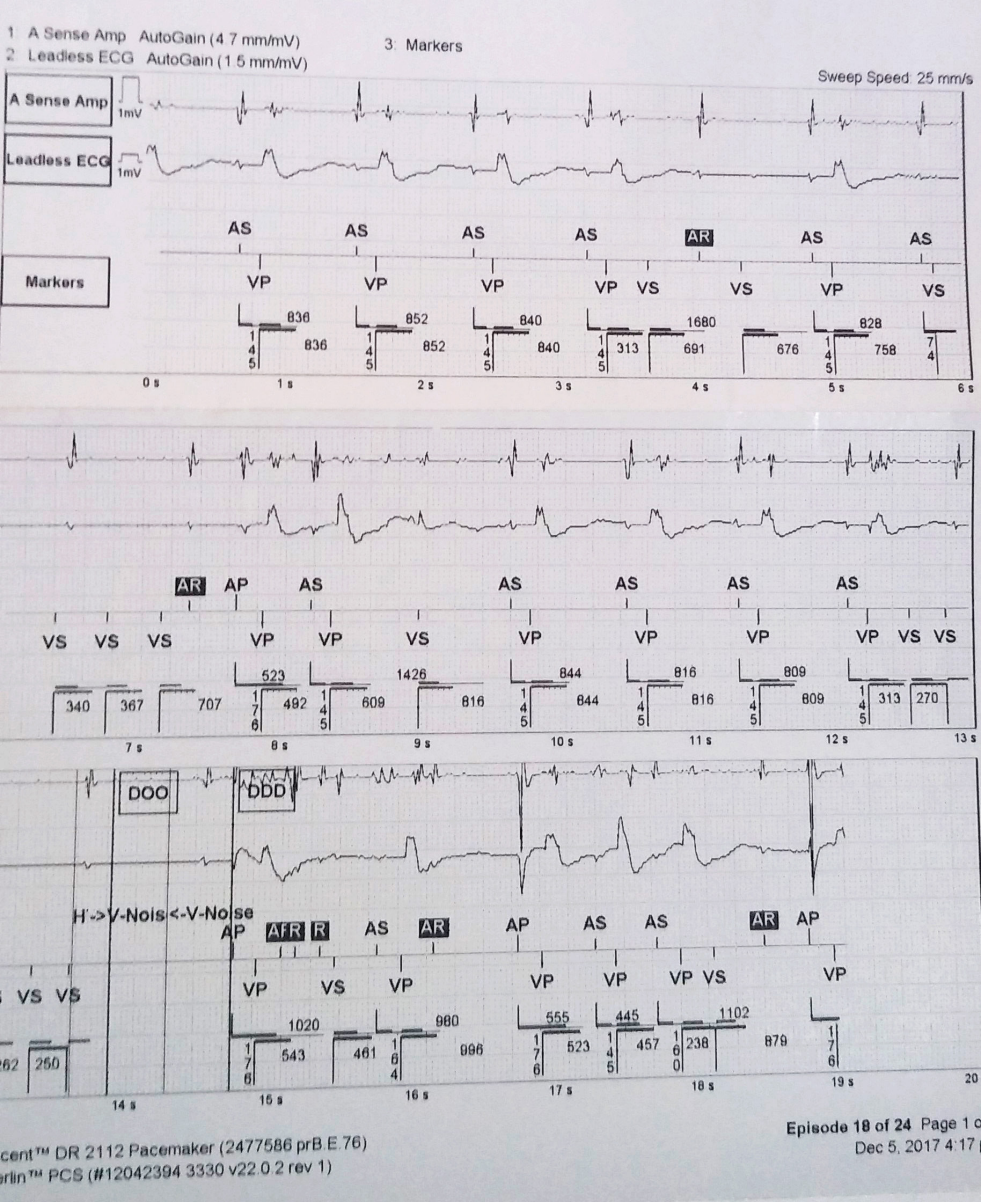
Incomplete electrode fracture. Pauses due to inhibitions of ventricular stimulation. ECG=electrocardiograms

Computerized PM analysis allowed us to determine the causes of syncope in 27.6% of the cases. Four cases (8.5%) occurred due to a command or sense failure. Three had a threshold increase, and one had an electrode fracture (Figure 1). Arrhythmias were recorded in eight cases with four cases experiencing SVT, three experiencing high rate AF, and one experiencing VF after acute coronary syndrome. One case of syncope occurred during an ST-segment elevation infarction with no record of concomitant arrhythmias.

The 24-hour Holter monitor was a useful diagnostic tool in 18.7% of the cases, with a diagnostic power of only 3%. The tilt table test was important for diagnosis in 28.5% of the cases, and the carotid sinus massage in one case. IES were performed in nine patients but provided inconclusive results.

## DISCUSSION

As demonstrated in the present study, the most frequent cause of syncope in PM patients appears to be neurally mediated^[3,4]^. Patients with an New York Heart Association (NYHA) Functional Class II or III, postural hypotension, and PM dependence may have a higher risk of syncope. Electronic evaluation of the PMs and the tilt table test were found to be the best methods to diagnose the causes of syncope.

### Causes of Syncope in PM Patients

In this study, there were varying causes of syncope observed, ranging from reflexes (vasovagal or drug-induced postural hypotension) to ventricular arrhythmias, acute myocardial infarction, and device dysfunction^[5]^. The causes of syncope found in this study are comparable with those described in the literature. Research conducted during the 1990s already showed a high prevalence of neurally-mediated syncope of unknown origin with PM dysfunction rarely being the cause (only 4.3% and 6.5%, respectively)^[9,10]^. Medeiros^[11]^ conducted a study during the 1980s that showed electrode and generator dysfunction as potential causes; however, device technology was different then. Newer studies also find that PM malfunction rarely causes syncope and affects approximately only 5% of patients^[12,13]^. When syncope occurs early after PM implantation, this should be the most relevant hypothesis with the cause identified after further investigation and computerized analysis of the device^[5]^.

In contrast, patients with old electrodes may present with complete or partial fractures that generate command failures or inhibitions (noises generated by electrode insulation damage) and exhibit prolonged pauses and low-output symptoms, such as dizziness, pre-syncope, or syncope. In this study, it was observed that most patients who presented with syncope had previous episodes of pre-syncope, and a detailed investigation by a professional should be considered.

It should be emphasized that reflexes may be aggravated by certain conditions, such as therapies used for arterial hypertension or heart failure leading to worsening OH, as well as the onset or progression of some pathologies including diabetes, renal failure, Parkinson's disease, cognitive disorders, or other degenerative neurological diseases^[3]^. Because of this, it is always necessary to consider patients’ drug use when diagnosing the cause of syncope^[3]^. PM should also be checked for defects, such as displacement in more recent implants or fractures in old electrodes, and the programming mode should be evaluated for underlying diseases^[3]^. Although a rare cause of syncope, pacemaker syndrome can also be a causative factor, and was unequivocally evidenced in one patient during our study.

### Predictive Factors for Syncope in PM Patients

Of the factors related to syncope, the only significant variable in the multivariate analysis was Functional Class II. Patients in Functional Class II had a nine-fold increased risk for syncope. However, according to univariate analysis, variables including Functional Class III, the presence of postural hypotension, and PM dependent patients without an escape rhythm may all indicate higher syncope risks. The group with syncope also exhibited a higher incidence of comorbidities, structural heart disease, higher levels of PM dependence, more advanced Functional Class, and may have had a higher rate of underlying diseases. Therefore, this population may inherently be at greater risk for syncope and any syncope-related consequences. Additionally, Functional Classes II and III patients have more severe disease and comorbidities.

A large group of patients (32%) did not exhibit prodromal symptoms, whereas 29.7% required hospitalization due to experiencing a high number of episodes over a short period of time, and 10.7% of the group with syncope presented with serious clinical findings, including physical trauma. It should be noted, however, that the syncope group and the control group did not differ in sex, age, ejection fraction, and left ventricular diastolic diameters. Therefore, the severity of the underlying cardiopathy did not justify the clinical occurrences.

The DANPACE study, one of the largest studies on syncope and PMs, found that the main risk factor for syncope after PM implantation was the occurrence of syncope prior to implantation^[12]^. This risk factor was found in 86% of the study group; however, there was a similar presentation in both the case and control groups. A causal factor with a plausible trigger for the clinical episode was discovered in 40.4% of patients with syncope. Therefore, it is important to observe and prevent certain actions that can lead to a greater risk for falls, pre-syncope, or syncope in elderly patients or patients with cardiopathy.

### Diagnostic Exams for PM Patients in Syncope Research

Of the tests used to investigate the causes of syncope, the tilt table test was the most promising. It diagnosed neurally-mediated syncope in approximately one-third of the cases. The tilt table test can also predict the chances of a patient experiencing recurrent reflex syncope^[3]^ with a high diagnostic capacity for PM patients who experience syncope and present with postural symptoms^[4]^.

The tilt table test results can be interpreted to show greater susceptibility to hypotension related to postural stress and are also capable of identifying individual tendencies toward syncope. This test can help identify a diagnosis, but there is no standard test to compare it with in order to provide true positive results. Therefore, obtaining information from the patient regarding episode similarities is also very important to confirm a diagnosis. We were able to confirm PM syndrome in one patient with a ventricular PM during the tilt table test when intermittent ventricular pacing with ventricular retrograde conduction was observed with concomitant symptoms of low cardiac output and hypotension. After changing the patient to a double-chamber system, a significant reduction in the number of episodes was observed despite incomplete elimination^[14]^.

The high diagnostic capacity of a PM analysis follows the evolution of several long-term monitoring techniques that have revolutionized the diagnosis of syncope and reduced unexplained syncope rates to < 20%. Current PMs have improved the morphology of their tracings, allowing for an adequate electrocardiographic analysis. It is crucial to analyze the tracings because the diagnoses suggested by the devices are not always correct and can induce errors. Medical professionals should remember to program the EGMs to detect relevant arrhythmias and perform regular analyses during follow-ups or symptomatic events.

The Holter monitor may detect intermittent command failures or inadequate inhibitions that have not been verified during programming tests. It is a cost-effective test; however, it has low diagnostic sensitivity. Some patients received a diagnosis after repeated 24-hour Holter monitoring or with a seven-day Holter monitor.

In this study, we chose to perform an IES in nine patients due to inadequate endocavitary records and clinical suspicion of syncope due to tachyarrhythmia; however, the results were negative in all of the patients. One patient had recurrent ventricular tachycardia and VF four days after elective PM replacement and was successfully defibrillated. In this case, there was no endocardial record of the arrhythmias, most likely owing to the low potential of the VF waves. This was one of the reasons we were motivated to perform IES in other cases. In this case, an emergency catheterization was performed to observe the sub-occluded right coronary artery, and an angioplasty was performed to stabilize the patient.

Patients with uni- or bicameral PM experiencing syncope require immediate evaluation by a cardiologist specialized in arrhythmias using a well-established clinical protocol. This should include an evaluation with a detailed clinical history, physical examination with supine blood pressure and orthostatic measurements, and PM analysis along with the stored EGMs. In certain cases, the diagnosis may only become apparent during clinical follow-up. This study group primarily included older patients with a high incidence of structural heart disease and comorbidities who are generally patients with high morbidity and mortality risks. The most frequent cause of syncope appears to be reflex syncope or dysautonomia^[15]^; however, life-threatening issues may also cause syncope in PM patients.

## CONCLUSION

The most frequent causes of syncope in PM patients were neurally mediated; however, there is a need for a more detailed investigation in this population because severe, life-threatening issues can also cause syncope. NYHA Functional Class II patients have an elevated risk of syncope. Patients with postural hypotension, NYHA Functional Class III, and PM dependence may also be at an increased risk for syncope. PM tests with an analysis of the stored EGMs and the tilt table test were the best methods to diagnose the causes of syncope.

**Table:** 

Authors' roles & responsibilities	
EAR	Investigation; final approval of the version to be published
GSC	Investigation; final approval of the version to be published
ABT	Investigation; final approval of the version to be published
ABVJ	Statistical analysis; final approval of the version to be published
ARPQ	Supervision; visualization; writing-review; final approval of the version to be published
FTMP	Investigation; final approval of the version to be published
MPMM	Investigation; final approval of the version to be published
MEQAR	Writing-review; final approval of the version to be published
CRFG	Investigation; final approval of the version to be published
CRMRS	Supervision; final approval of the version to be published

## References

[1] Brignole M, Moya A, de Lange FJ, Deharo JC, Elliott PM, Fanciulli A (2018). 2018 ESC guidelines for the diagnosis and management of syncope. Eur Heart J.

[2] Shen WK, Sheldon RS, Benditt DG, Cohen MI, Forman DE, Goldberger ZD (2017). 2017 ACC/AHA/HRS guideline for the evaluation and management of patients with syncope: executive summary: a report of the American college of cardiology/American heart association task force on clinical practice guidelines and the Heart rhythm society. J Am Coll Cardiol.

[3] Sutton R (2015). Syncope in patients with pacemakers. Arrhythm Electrophysiol Rev.

[4] Haarmark C, Kanters JK, Mehlsen J (2015). Tilt-table testing of patients with pacemaker and recurrent syncope. Indian Pacing Electrophysiol J.

[5] Bhargava K (2015). Tilt test in paced patients: Is it worth the effort?. Indian Pacing Electrophysiol J.

[6] Sumiyoshi M (2014). Role of permanent cardiac pacing for vasovagal syncope. J Arrhythm.

[7] Sutton R, Brignole M (2014). Twenty-eight years of research permit reinterpretation of tilt-testing: hypotensive susceptibility rather than diagnosis. Eur Heart J.

[8] Santini M, Castro A, Giada F, Ricci R, Inama G, Gaggioli G (2013). Prevention of syncope through permanent cardiac pacing in patients with bifascicular block and syncope of unexplained origin: the PRESS study. Circ Arrhythm Electrophysiol.

[9] Pavlovic SU, Kocovic D, Djordjevic M, Belkic K, Kostic D, Velimirovic D (1991). The etiology of syncope in pacemaker patients. Pacing Clin Electrophysiol.

[10] Sgarbossa EB, Pinski SL, Jaeger FJ, Trohman RG, Maloney JD (1992). Incidence and predictors of syncope in paced patients with sick sinus syndrome. Pacing Clin Electrophysiol.

[11] Medeiros P (1989). Syncopes and dizziness in patients with cardiac pacemakers. Relampa.

[12] Ng Kam Chuen MJ, Kirkfeldt RE, Andersen HR, Nielsen JC (2014). Syncope in paced patients with sick sinus syndrome from the DANPACE trial: incidence, predictors and prognostic implication. Heart.

[13] Ofman P, Rahilly-Tierney C, Djousse L, Peralta A, Hoffmeister P, Gaziano JM (2013). Pacing system malfunction is a rare cause of hospital admission for syncope in patients with a permanent pacemaker. Pacing Clin Electrophysiol.

[14] Arif A, Khan R, Tran N (2017). Pacemaker syndrome; an often overlooked diagnosis in patients with pacemakers. J Mol Cell Cardiol.

[15] Yasa E, Ricci F, Holm H, Persson T, Melander O, Sutton R (2019). Cardiovascular autonomic dysfunction is the most common cause of syncope in paced patients. Front Cardiovasc Med.

